# Supplementary material for: Emerging carbapenem-resistant Klebsiella pneumoniae in a tertiary care hospital in Lima, Peru
Source: Microbiol Spectr. 2025 Jan 10;13(2):e01825-24. doi: 10.1128/spectrum.01825-24 (PMC11792469; doi:10.1128/spectrum.01825-24)
Supplement: Supplemental material — Fig. S1 to S9; Table S1. [file spectrum.01825-24-s0001.docx]

**SUPPLEMENTAL MATERIALS**

Figure S1. Description of the three sets of CRKP isolates included in the study. The number of isolates recovered at the clinical microbiology laboratory of a tertiary care hospital in Lima, Peru and regrown at the IMTAVH research laboratory is shown.


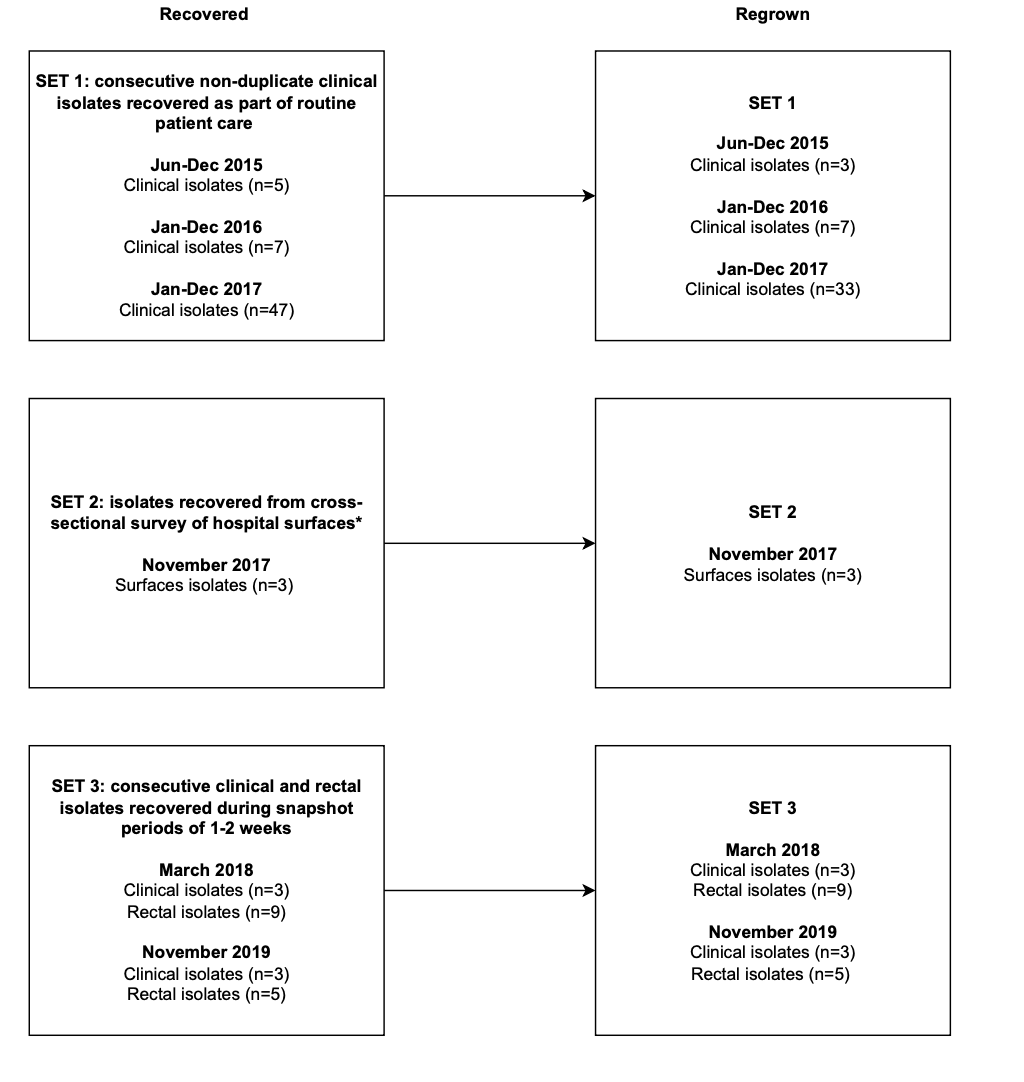


*Cross-sectional survey of hospital surfaces conducted by the Hospital’s Epidemiology Office.

A survey of high-touch surfaces was conducted the 30th of November 2017 by personnel of the Hospital’s Epidemiology Office at 6 different services of the hospital: Intensive care unit, intermediate care unit, surgical intensive care unit, trauma & shock unit of the emergency department, and three operating rooms)

Samples were collected from pre-specified high-touch surfaces at each service, using a sterile cotton swab. After swabbing the surface, the cotton swab was placed on a tube with TSB named and stored in a cooler. All samples were transferred to the laboratory within 4 hours. At the laboratory, inoculated TSB broth was incubated overnight for 18 hours at 37ºC. The overnight growth was then inoculated in MacConkey Agar and incubated for 18 hours at 37ºC. Biochemical identification was then performed to all isolates recovered and susceptibility to meropenem and ceftriaxone was tested using disk diffusion method according to CLSI (2017) guidelines.

A total of 61 surfaces were sampled. Of these, 9 samples presented growth, of which only three were identified as carbapenem-resistant *K. pneumoniae*. These three positive samples were from:

- Bed rail at the surgical ICU
- Ventilator at the surgical ICU
- Isolation stethoscope at the medical ICU

The other 6 positive surface cultures were identified as *Enterobacter cloacae* (4) and carbapenem-susceptible *K. pneumoniae* (1).

Figure S2. Pairwise SNP distance between *Klebsiella* *pneumoniae* sequences (n=50). Distribution of SNPs between (A) all pairs of *K.* *pneumoniae* sequences, (B) capped at 100 SNPs and (C) within the four most frequent ST groups. Figures were obtained using GraphSNP online tool.

A


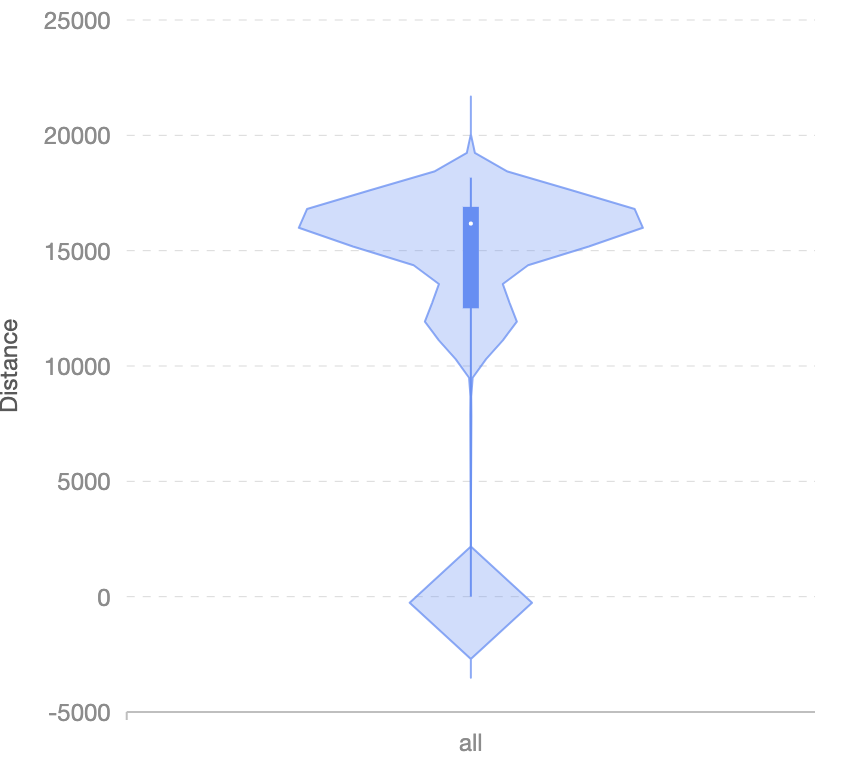


B


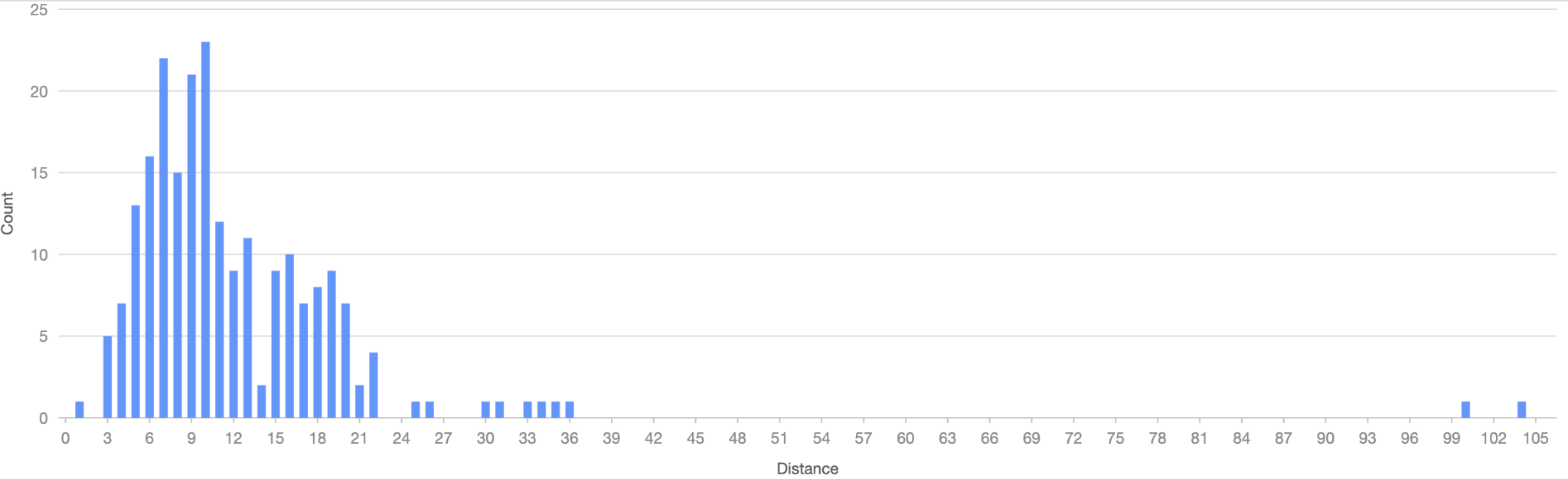


C


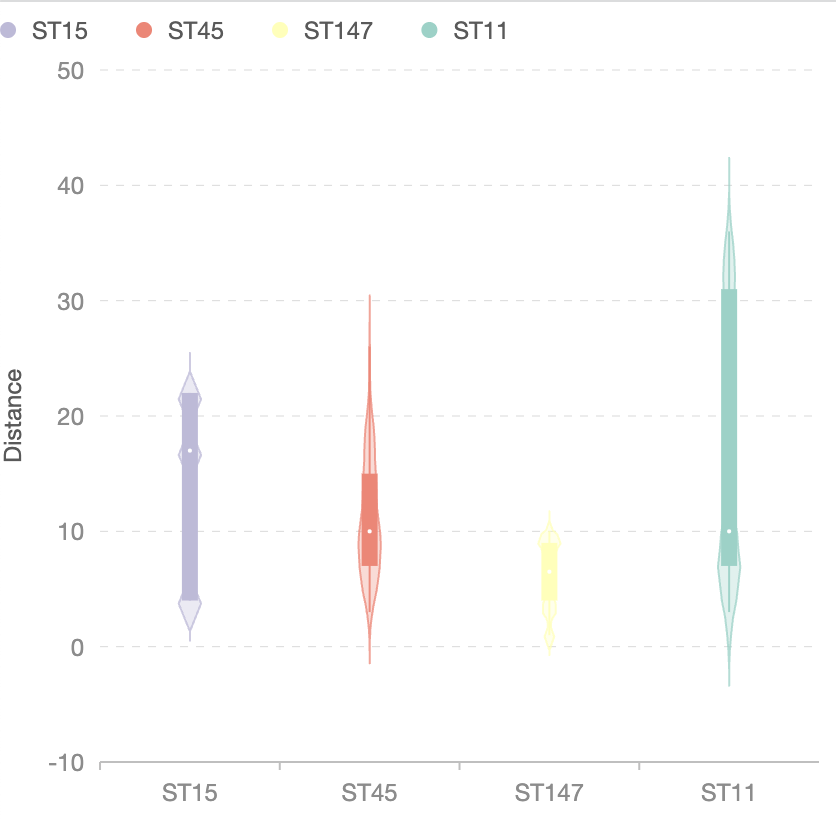


Figure S3. Putative clusters identified among *Klebsiella* *pneumoniae* sequences (n=50) using a (A) threshold of ≤10 SNPs and (B) threshold of ≤15 SNPs. Red numbers represent the SNP distances, all SNP distances ≤10 are presented with a line. Node colors represent the ST groups. Figures were obtained using GraphSNP online tool (ref).


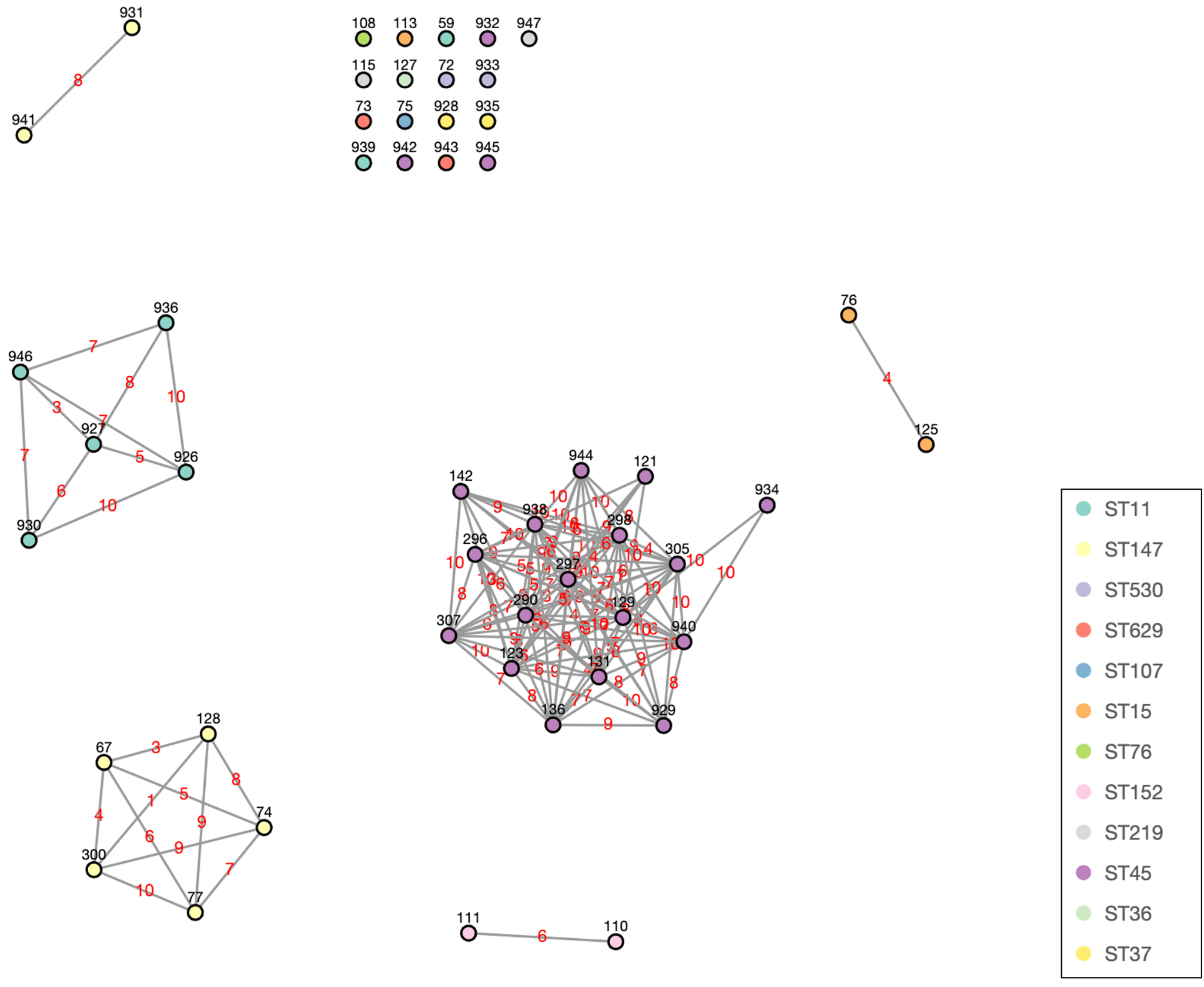


Figure S4. Cluster analysis of the largest cluster including 17 ST45 *Klebsiella* *pneumoniae* sequences. SNP distances to the genetically closest neighbor ≤ 10 SNPs are presented with a full line. Dotted lines represent SNP distances ≤ 5 SNPs sequences that are not the genetically closest neighbor. Red numbers represent the SNP distances. Node colors represent the hospital ward.


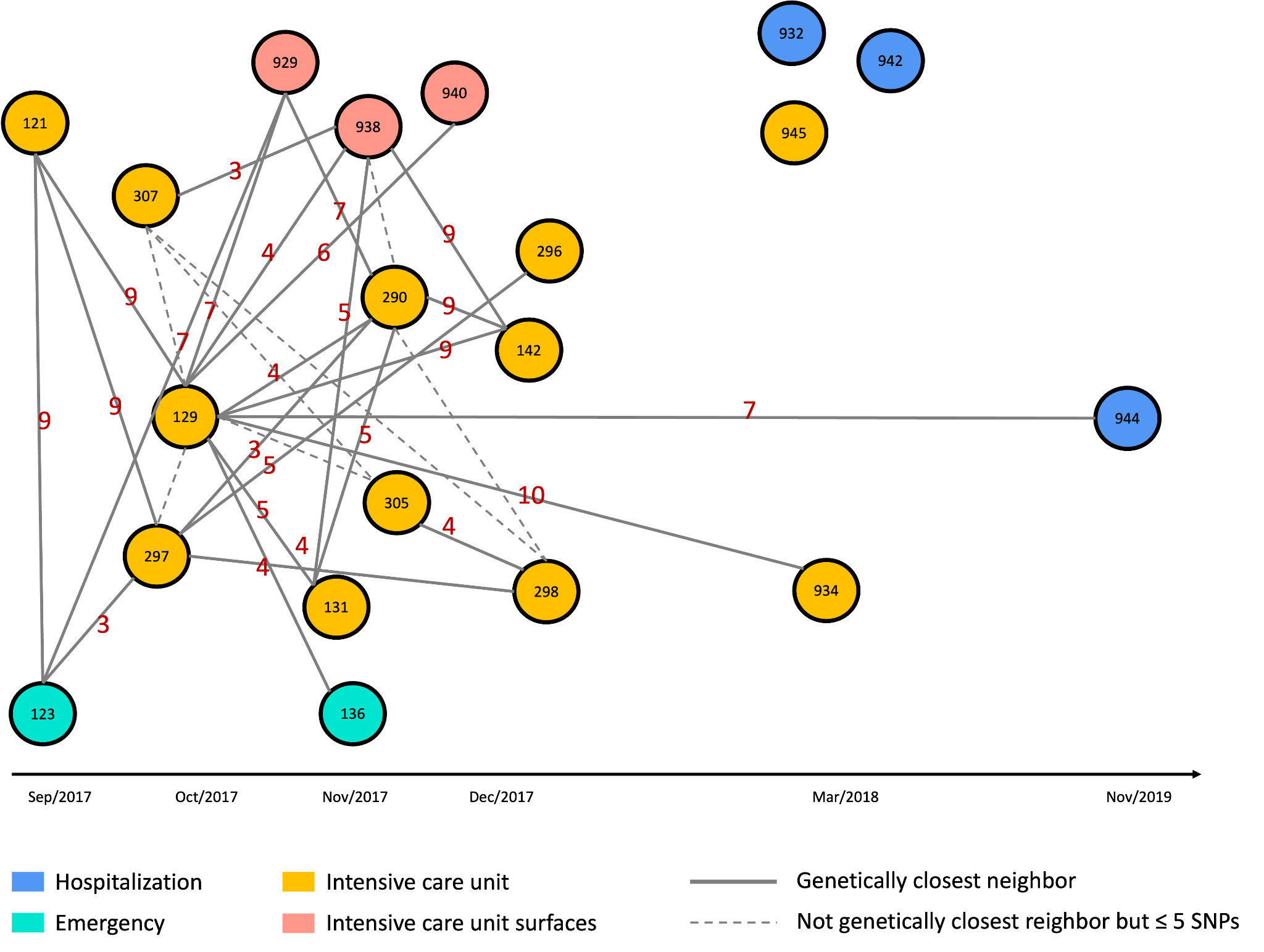


Figure S5. Gene synteny in assemblies containing (A) blaKPC-2 and (B) blaNDM-1 carbapenemases. Genes are annotated by predicted function: antibiotic resistance (red), association with horizontal gene transfer (green), other ORFs (yellow). Only genes larger than 700bp are shown. Shading denotes areas of 100% similarity across genomes.


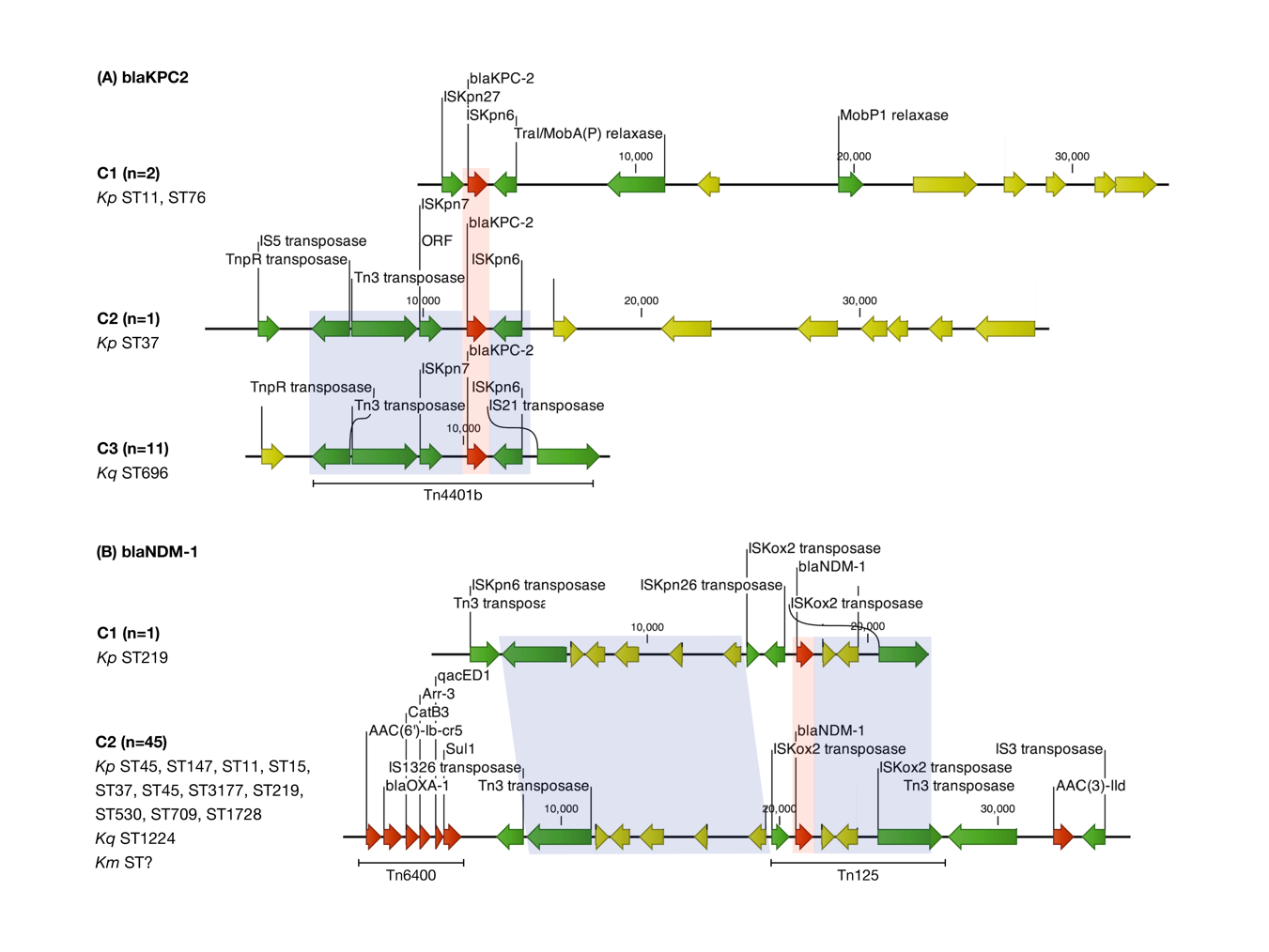


Figure S6. Systematic search of publicly available carbapenem-resistant *K. pneumoniae* sequences from South American countries uploaded in the NCBI Pathogen Detection Database from January 2000 to November 2022.


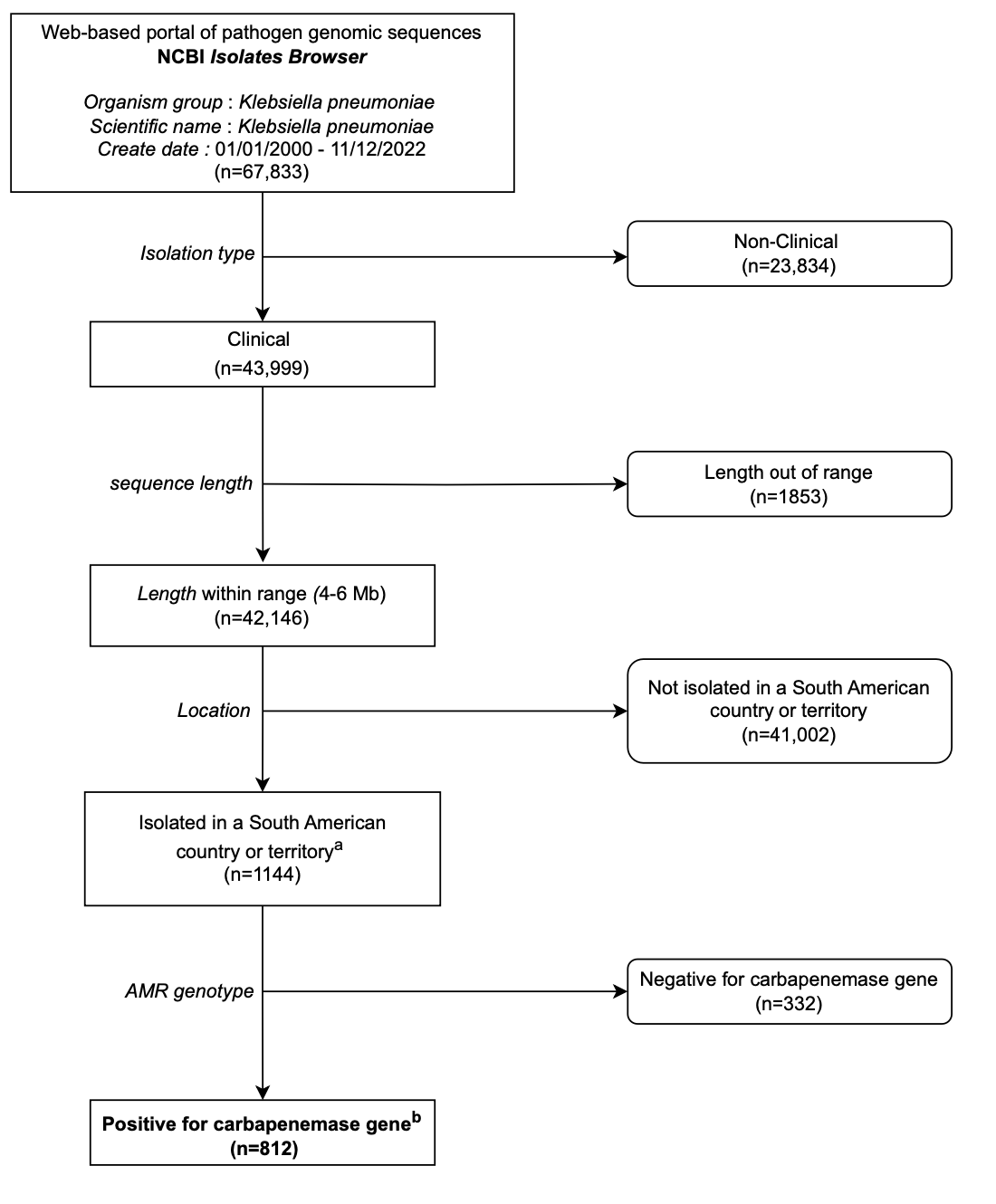


^a^South American countries or territories with at least one sequence available: Argentina, Brazil, Chile, Colombia, Ecuador, Paraguay, Peru, Trinidad and Tobago, Uruguay, Venezuela.

^b^AMR genotype available: *bla*_KPC_, *bla*_NDM_*, bla*_OXA-181-like_*, bla*_IMP_*, bla*_VIM_

Figure S7: Systematic search of published studies reporting carbapenem-resistant *K. pneumoniae* whole-genome sequencing in South America.


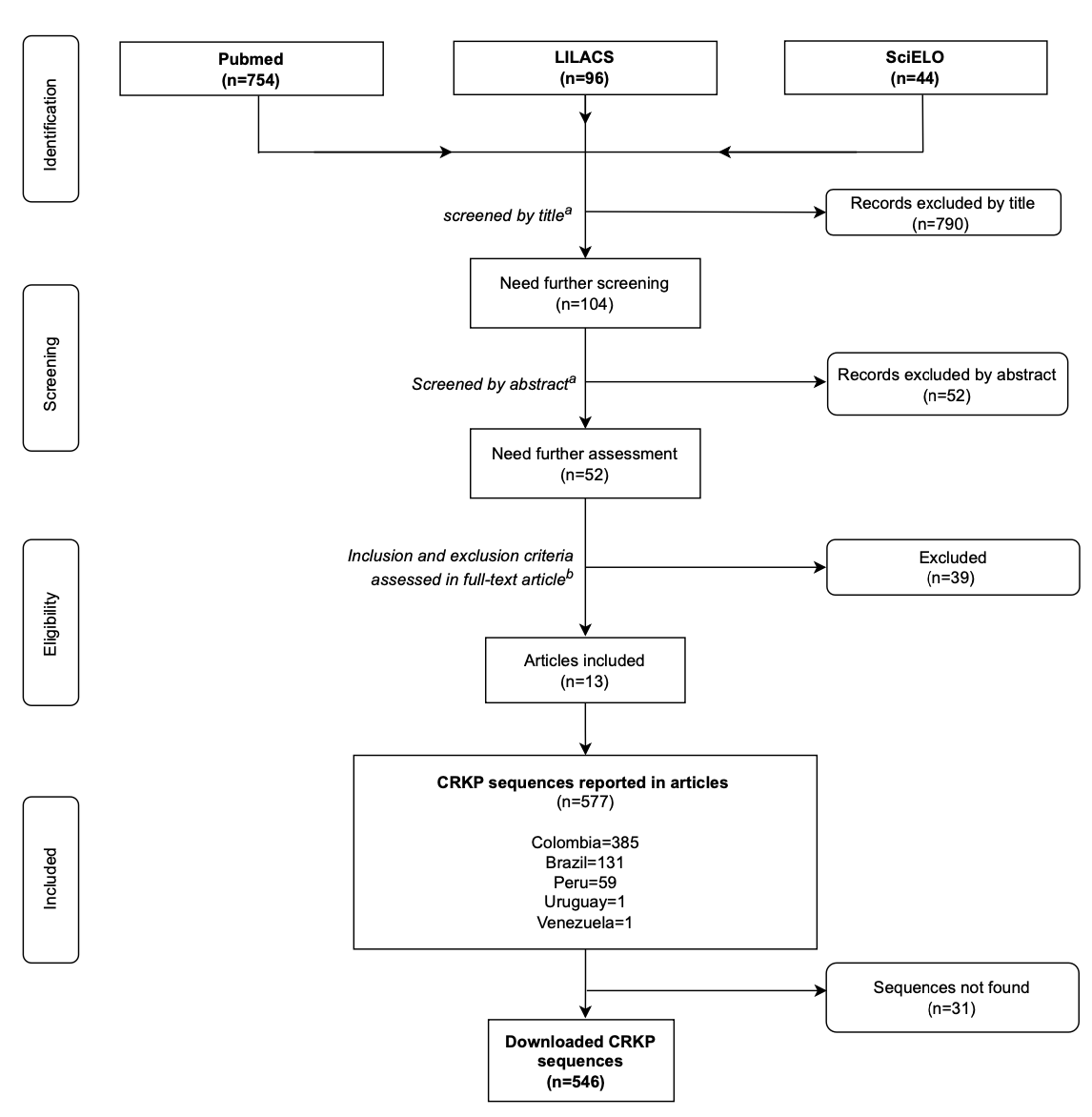


^a^Titles and abstracts that did not mention *Klebsiella pneumoniae* or were from a non-South American country were excluded.

^b^Inclusion criteria: Original article, written in English, Spanish or Portuguese, published between 2000-2022, reported whole genome sequencing of a carbapenem-resistant *Klebsiella pneumoniae* isolated in any South American country or territory.

For the search in PubMed, we used the following MeSH terms and entry terms: (((#1) AND (sequencing)) OR ((#1) AND (genome)) OR ((#1) AND (carbapenemase)) OR ((#1) AND (carbapenem resistance)), where #1 was *K. pneumoniae*. We excluded meta-analyses, books, and systematic reviews. For the searches in SciELO and LILACS, the terms “*Klebsiella pneumoniae* resistente carbapenémicos” OR “*Klebsiella pneumoniae* carbapenemasa” were used, with no additional filters. All articles were then manually reviewed to evaluate if they met the following inclusion criteria: Publication date between 2000-2022; English, Spanish, or Portuguese language; contained results from the genomic sequencing of CRKP clinical isolates recovered in any of the 14 countries or territories of South America within the period January 2000 to December 2021.

Figure S8. Quality assessment of South American carbapenem-resistant *K. pneumoniae* sequences found through the two systematic searches to include high-quality sequences in the maximum-likelihood tree.


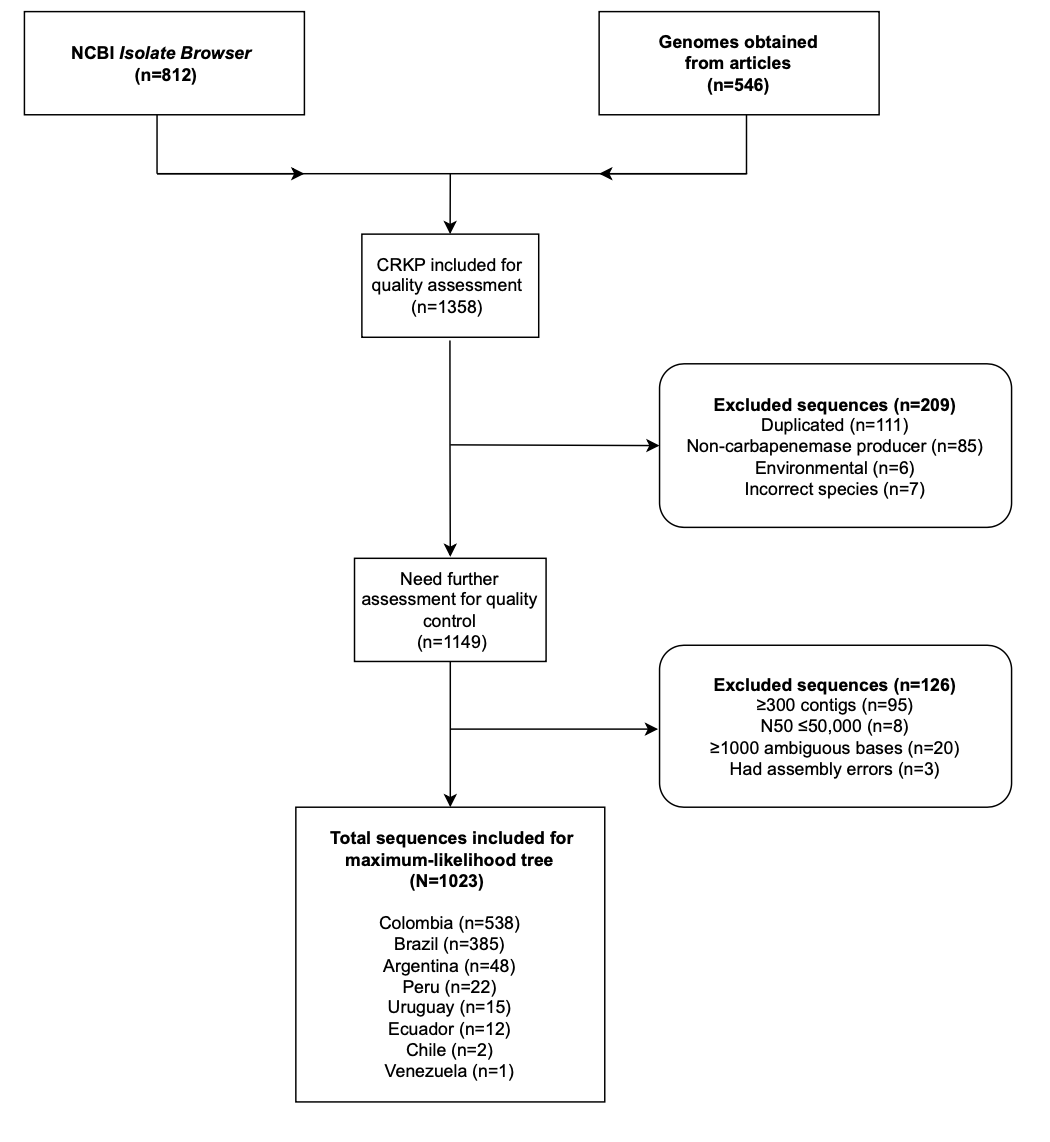


Figure S9. Distribution of ST groups of (A) Peruvian carbapenem-resistant *K. pneumoniae* sequences compared with (B) sequences from other South American countries. Figures were obtained using KleborateVIZ online tool.

A


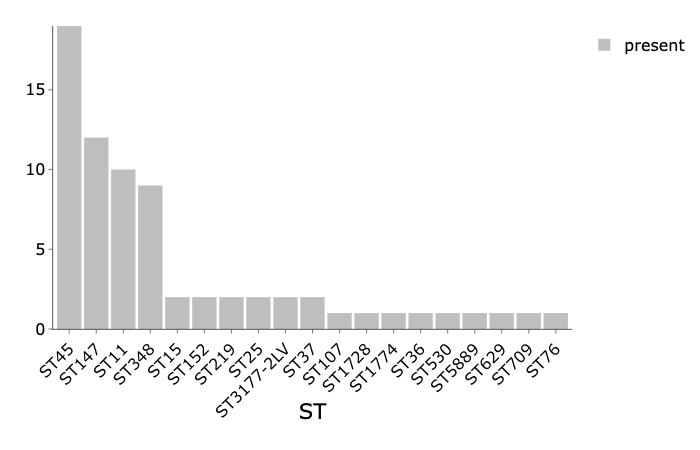


B


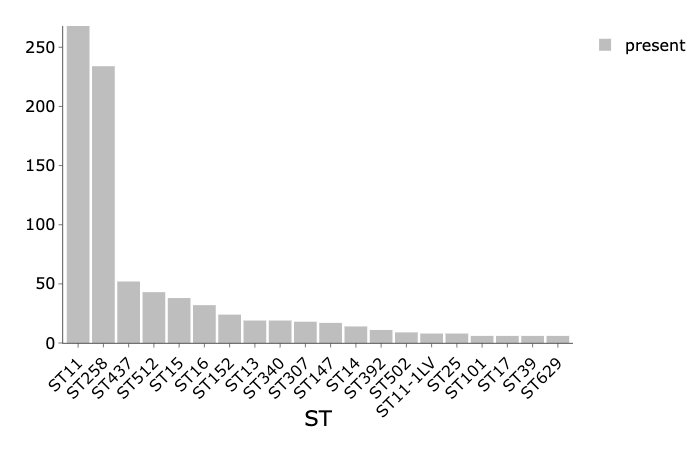


Table S1. Genetic determinants of carbapenem resistance and virulence in carbapenem-resistant *K. pneumoniae* sequences of Peru and other South American countries.

| Genomic determinants | Total  (N=1071) | | Peru  (N=70) | | Other countries  in South America  (N=1001) | |
| --- | --- | --- | --- | --- | --- | --- |
|  | n | (%) | n | (%) | n | (%) |
| Carbapenemase genes |  |  |  |  |  |  |
| *bla*_KPC-2_ | 599 | (55.9) | 4 | (5.7) | 595 | (59.4) |
| *bla*_KPC-3_ | 265 | (24.7) | 0 | (0.0) | 265 | (26.5) |
| *bla*_KPC-30_ | 2 | (0.2) | 0 | (0.0) | 2 | (0.2) |
| *bla*_NDM-1_ | 150 | (14.1) | 57 | (81.4) | 93 | (9.3) |
| *bla*_NDM-5_ | 2 | (0.2) | 0 | (0.0) | 2 | (0.2) |
| *bla*_IMP-16_ | 5 | (0.5) | 5 | (7.1) | 0 | (0.0) |
| *bla*_VIM-2_ | 13 | (1.2) | 0 | (0.0) | 13 | (1.3) |
| *bla*_VIM-24_ | 2 | (0.2) | 0 | (0.0) | 2 | (0.2) |
| *bla*_VIM-4_ | 7 | (0.7) | 0 | (0.0) | 7 | (0.7) |
| *bla*_OXA-48_ | 0 | (0.0) | 0 | (0.0) | 0 | (0.0) |
| *bla*_OXA-181_ | 3 | (0.3) | 3 | (4.3) | 0 | (0.0) |
| Dual carbapenemase | 22 | (2.1) | 1 | (1.4) | 21 | (2.1) |
| Triple carbapenemase | 1 | (0.1) | 0 | (0.0) | 1 | (0.1) |
| Virulence loci |  |  |  |  |  |  |
| Capsular serotype |  |  |  |  |  |  |
| K1 | 1 | (0.1) | 0 | (0.0) | 1 | (0.1) |
| K2 | 21 | (2.0) | 0 | (0.0) | 21 | (2.1) |
| Siderophores |  |  |  |  |  |  |
| *ybt* | *580* | (54.2) | 40 | (57.1) | 540 | (53.9) |
| *clb* | *261* | (24.4) | 0 | (0.0) | 261 | (26.1) |
| *iuc* | *9* | (0.8) | 6 | (8.6) | 3 | (0.3) |
| *iro* | *0* | (0.0) | 0 | (0.0) | 0 | (0.0) |
| Mucoid phenotype regulator |  |  |  |  |  |  |
| *rmpADC* | *1* | (0.1) | 0 | (0.0) | 1 | (0.1) |
